# Resuming work roles after injury in a low-income context: Multiple factors influencing the return to work outcomes

**DOI:** 10.1371/journal.pone.0308816

**Published:** 2024-10-23

**Authors:** Ansha Nega Ahmed, Marcia Finlayson, Adamu Addissie, Ayalew Zewdie, Rosemary Lysaght

**Affiliations:** 1 School of Rehabilitation Therapy, Queen’s University, Kingston, Ontario, Canada; 2 School of Public Health, Addis Ababa University, Addis Ababa, Ethiopia; 3 Addis Ababa Burn, Emergency and Trauma Hospital, St. Paul’s Hospital Millennium Medical College, Addis Ababa, Ethiopia; Royal College of Surgeons in Ireland, IRELAND

## Abstract

**Background:**

Return to work (RTW) is an important outcome indicator for the effectiveness of rehabilitation services, and the functional status and overall recovery among individuals who have experienced injury. Despite the rising incidence of traumatic injury among economically productive citizens in Ethiopia, there is a paucity of evidence about the RTW status of injury survivors. This study examined factors associated with RTW success and determinants of time to RTW after injury in Ethiopia.

**Methods:**

An institution-based cross-sectional study was conducted to collect data retrospectively one year after patients arrived at the study setting due to traumatic injuries. Medical records of all patients who visited the emergency room of a large public hospital in Addis Ababa were reviewed. Data were collected from survivors of traumatic injury, interviewed by telephone one year post-injury. Multivariable logistic regression and survival analysis were carried out to explore factors.

**Results:**

Of the 251 participants, 75% were young adults (age </ = 39 years), 78% were male, 78% were urban residents, 41% were injured by road traffic collisions, and 59% returned to work within one year. The logistic regression model revealed *short inpatient admission* (AOR = 4.20; 95% CI: 2.10–8.50; *p* ≤ 0.001), *no disability* (AOR = 4.44; 95% CI: 2.10–9.60; *p* ≤ 0.001), *motivation to RTW* (AOR = 3.50; 95% CI: 1.61–7.50; *p* = 0.002), *no chronic illness* (AOR = 2.31; 95% CI: 1.14–4.70; *p* = 0.020), being in an *administrative position* (AOR = 5.32; 95% CI: 1.11–25.78; *p* = 0.038) and *receiving injury compensation* (AOR = 3.10; 95% CI: 1.22–7.73; *p* = 0.017) as factors for successful RTW within a year after injury. Further, the Cox regression analysis identified *immediate access to healthcare after injury* (AHR = 1.54; 95% CI: 1.05–2.25; *p* ≤ 0.026) and having injury of penetrative to internal organ, *strain*, *sprain*, *dislocation or soft tissue* (AHR = 1.81; 95% CI: 1.20–2.80; *p* = 0.007) as determinants of early RTW after traumatic injury.

**Conclusion:**

The study uncovers factors crucial to RTW planning and interventions, and provides insights to minimize barriers, foster a smooth transition to employment, and optimize survivors’ lives after injury.

## Introduction

Return to work (RTW) is an important marker for an individual’s recovery from traumatic injury, the effectiveness of rehabilitation services for residual effects, and functioning in the real world [1, 2]. Work is frequently reported as one of the key social determinants of human health and as having a central role in an adult’s life [3, 4]. Those who lack work opportunities may experience multifaceted consequences, such as negative impacts on overall well-being, including financial, psychological, and physical wellbeing. In light of these consequences, employment has the potential to support improvements in physical and psychosocial health after life-changing health problems, including injuries [5]. A recent study in the UK [6] reported on the importance of RTW after traumatic injury to enhance survivors’ sense of purpose in life, nurture self-identity, and ensure social interaction. Therefore, failing to resume work after injury—in addition to the financial setbacks both on the injured worker and employer—could lead to serious consequences for the psychological and physical well-being of individuals [7].

A RTW program is a comprehensive rehabilitation effort composed of multidimensional interventions, which maximize individuals’ functioning and manage residual consequences of health conditions, in this case, traumatic injury [8, 9]. RTW is not just an event, but rather a dynamic process that may be iterative [10, 11], such that the return may be interrupted one or more times by various factors, or work hours may fluctuate during the reintegration process. It also involves various changes throughout the recovery process: physical recovery, motivation, behaviour, and interaction in the social system [11]. Because of its dynamic nature, designing a RTW strategy and evaluating the outcomes by stages of recovery after injury are important.

The success of RTW after work disability includes the concepts of individuals’ employment status, type of work, time to return, and the sustainability of RTW [2, 8]. The importance of early RTW after injury and illness has been documented in the literature as it is beneficial to managing health conditions, improving individual well-being, and reducing impacts on workplaces and society [12, 13]. In addition to the static employment outcome, some studies measure time to RTW after injuries or illnesses [8, 14], for example, within three months or within a year after the injury. The longer the person stays off work, the lower the probability of returning to work and the sustainability of the RTW [15–18]. Despite the benefit of early RTW, careful consideration of associated risks is crucial. The potential risks related to a RTW that is too soon may include re-injury due to insufficient recovery or secondary injury that results when the person changes the mechanism of task performance to accommodate ongoing pain. There may also be psychological problems of anxiety and frustration associated with reduced work performance [12, 19].

The RTW success of injury survivors is determined by various factors due to interactions between multistage actors in a complex biopsychosocial system [20, 21]. Many factors are known to influence return-to-work outcomes of injury survivors, including personal factors (e.g., age, sex, education and residence); injury characteristics and associated effects (e.g., severity, cause, type and resulting impairment); and employment characteristics (e.g., types of jobs and sectors) [1, 20].

In Ethiopia, the employer is required by the law to provide job security and supports for injured workers up to one year after the injury, including wage adjustment and various other provisions. For example, employers will continue to pay 100% of the injured worker’s regular salary for the first three months; the amount is reduced by 25% after 3 months of absence and to 50% for the following 6 months. In addition, employers have an obligation to provide injury compensation, including the cost of short-term needs (e.g., medical expenses) [22, 23]. Employers could absorb these costs fully or partially by arranging workers’ insurance coverage.

Further, the employment injury compensation system is not governed by one legislation or a single administration. The Ministry of Labour and Social Affairs produced a National Social Protection Policy with the aim of improving social justice, supporting industrial peace, reducing poverty, and enhancing development. As a result, three laws are functional governing employment injury compensation: 1) Labour Proclamation No 1156/2019 [22], 2) Private Organization Employees Pension Proclamation No. 1268/2022 [24], and 3) Public Servants’ Pension Proclamation No. 1267/2022 [25]. In addition, employer liability coverage is also available through many private insurance companies for commercial operations. The government is interested in expanding social protection schemes to the informal sectors [26], and employees of informal sectors are covered under the Private Organizations Employees’ Pension Proclamation [24]. Nevertheless, a report on mapping Ethiopia’s employment injury compensation system indicates low compliance of employers, inadequate employment injury benefits and a practice of going away from employer-liability schemes [23]. Social security organizations are the final payers after employers and insurance companies and cover only long-term benefits (i.e., disability benefits) when a worker fails to resume work after injury. Further, available legal frameworks do not provide injury compensation coverage for international/diplomat workers, domestic workers, and self-employed individuals. Legal frameworks have a gap in indicating the responsible agent for and guiding the kinds of obligations employers have in supporting the RTW of injured workers.

Past research has conceptualized RTW as either an outcome or a process [8, 27], which depends on the focus of the researcher. Successful RTW outcome after an injury can be measured either by time to resume working after injury [8, 14] or as employment status, as indicated by job type, employer (same/different) or conditions (contract or wage) [8, 28]. RTW as a process often aims to explore ‘how’ individuals managed to return to work after the injury, which may include access to support, decisions, and actions along the journey to resume work post-injury [8, 29]. However, in Ethiopia, evidence on RTW outcomes and the determining factors are limited as they have received little attention on the research agenda and in service provision.

In this study, RTW is conceptualized as an outcome, specifically the participant’s employment status one year after injury, defined here as success in resuming work in any type of job, with any employer and at any capacity [30, 31]. The aim of the current study was to identify factors associated with successful RTW one year post-injury and to examine determinants of time to RTW after injury in Ethiopia, which is a low-income context.

## Methods and materials

### Study design and setting

An institution-based cross-sectional study was conducted to collect data retrospectively one year after patients arrived at the Addis Ababa Burn, Emergency and Trauma (AaBET) hospital due to traumatic injuries. The hospital was selected purposefully, with the aim of targeting an institution that specializes in trauma care. It is the largest trauma center in Addis Ababa, where most people go to seek healthcare services after a traumatic injury [32].

### Study population

The study population included individuals with a record in the Health Management Information System (HMIS) and charts of individuals who were initially admitted at the study setting during the period October 11, 2020, to January 8, 2021. The inclusion criteria for participant eligibility were the initial hospital admission due to injury, being a working-age adult (18 to 60 years), being discharged alive, being admitted in the hospital for >/ = 24 hrs, self-identified as employed at the time of the injury and having functional telephone numbers.

### Participant recruitment procedure

Two stages of participant selection were carried out from two separate sources of patients’ medical records from December 15, 2021, to January 15, 2022. First, the first author screened the HMIS records to identify eligible patients. A total of 2042 patients were registered in the HMIS during the study period. The hospital registered the HMIS data on an Excel spreadsheet. The HMIS dataset includes information such as Medical Registration Number (MRN), age, sex, health conditions for hospital visits, date of arrival and discharge, and status at discharge.

In the second stage, 638 patient records were identified using the inclusion criteria. The variables extracted from the charts were mainly related to the injury characteristics and participants’ names and telephone numbers. Two trained health professionals extracted the data from patients’ charts, using a structured checklist, S1 Checklist.

Following the data extraction, the eligibility of potential participants was confirmed by telephone, and those eligible were invited for an interview. Of the 638 participants who fulfilled the inclusion criteria, 330 confirmed to participate in the study once contacted. As an example of the exclusion criteria, their employment status at the time of the injury was checked. If they were employed, their willingness to participate in the study was confirmed at this point. If they were not working, they were excluded from the study. Of the 330 eligible survivors, 76 were not interviewed for the following reasons: 14 declined to participate, and 62 could not be reached [33]. Finally, 254 injury survivors verbally consented to participate, and the telephone interview was conducted from January 20 to February 28, 2022. Of the 254 survivors, three participants didn’t complete the full interview; hence, they are excluded from this analysis.

### Telephone survey data collection procedure

Six trained health professionals conducted the telephone interview using a structured questionnaire, S1 File. Interviews were conducted in either Amharic or Afaan Oromo, based on participants’ preferences. The telephone interview included tailored questions designed for this study. The questionnaire development was informed by the Systemic, Ecological Model for Rehabilitation Counseling [20] and other literature in the field [8, 28, 31] to capture micro-to-macro level interactions of the injury survivors with various systems in the RTW process. The ecological model has four systems: the consumer system (e.g., injury survivor), the functional system (e.g., living and working system), the provider system (e.g., healthcare and rehabilitation service), and contextual systems (i.e., socio-cultural and economic conditions) that collectively influence individuals’ RTW experience. Hence, the questionnaire included information about sociodemographic factors, employment characteristics, and health-related information (e.g., disability state and chronic illness).

### Outcome variables

The main outcome of interest in this study is successful RTW, which is the RTW status of a participant one year after the injury, which was defined based on participants’ responses to the question “Are you currently returned to any work?”. The response options are “yes” or “no” for their RTW status at the time of the interview, to any job, any employer and with any capacity to work. In addition, the time to RTW was captured during interviews as a continuous variable, measured in weeks. Timely RTW is assumed if the person resumes work within 12 weeks, during which the individual would receive a full salary while on sick leave [22].

### Independent variables

#### Socio-demographic factors

Participant biological sex and age are captured as recorded on patients’ charts, and biological sex was grouped into male and female. Age was categorized into three groups: young workers (<25 years), young adults (25–39 years) and older adults (>39 years). Participants’ educational status, residence, marital status and living arrangement data were captured during the telephone interviews. Educational status was the highest grade a participant completed and was represented by four groups: no formal schooling, primary school (Grade 1^st^-8^th^), high school (Grade 9^th^-12^th^) and college and above. Participants’ residence was dichotomized into urban (at and above district administration, with >/ = 2000 inhabitants) and rural (below the level of district administration, with <2000 inhabitants) [34]. Marital status was categorized into single, married, divorced and widowed. Participants’ living arrangement was captured via the number of persons living in the same house and grouped into living alone and living with another person.

#### Employment characteristics

During the interviews, participants were asked a series of structured and open-ended questions to understand their pre-injury employment and post-injury employment conditions. During analysis, the responses were grouped into meaningful categories as follows: employer (government, private and self/family business); employment relationship (with contract and without contract); job types/position (manual/blue collar and managerial/white collar); and workplace size (micro and small enterprises (up to 30 workers) and medium and large-scale enterprises (> 30 workers)).

#### Injury characteristics

Information about injury characteristics was extracted from medical charts according to the International Classification of Diseases, 10^th^ revision (ICD-10) [35] and grouped into meaningful categories during analysis. Accordingly, mechanism of the injury was categorized into road traffic collisions, falling, violence, burn and electrocution and contact with external forces such as machine injury. The nature of injury was grouped into traumatic brain injury (TBI), fracture, and injuries of penetrative, soft tissue, or sprain/strain/dislocation. The body parts injured were grouped to the closest regions of the body and categorized as head, extremities (lower and/or upper), chest and internal organ, spine and bones of the trunk, and others.

#### Post-injury conditions

The number of days passed from the event of the injury to the arrival at the hospital and the length of inpatient stay, a proxy measure of injury severity, were extracted from the medical charts as continuous variables. The days from the injury occurrence to hospital arrival were categorized as “the same day” and “after a day or more.” Also, the number of the length of inpatient stay was categorized as “up to one week” and “more than a week.” Participants’ disability status was determined using the WHODAS-2, the 12-item assessment tool [36, 37], which inquires about the severity of the restrictions experienced in activity and participation, using a five-point Likert scale (from 0 = no restriction to 4 = very severe restriction). The tool had previously been translated to Amharic and validated for cultural adaptability [36]. The sum of all items was determined with a minimum score of ‘0’ and a maximum score of ‘48’. The values were categorized into: <6 = no significant restriction; 6–11 = mild restriction; 12–24 moderate restriction; 25–36 = severe restriction; and 37–48 = very severe disability or complete restriction. The presence of chronic illnesses at the time of the interview (as diagnosed by a healthcare professional) was assessed based on participants’ responses as “yes” or “no.”

Injury compensation was captured during interviews and grouped into received and not received. Moreover, participant motivation to RTW even before full recovery was captured with a question of “Would you prefer to go to/show up at your workplace, even though you are not fully recovered, or your health condition is not good enough to the extent it compromises your productivity?”. It has a five-point Likert scale response (i.e., from always to never) where the responses were grouped into ‘*no motivation*’ if the response is never and *‘have motivation’* for all other than never. Work-related stressors experienced by injury survivors in the RTW process were captured during interviews with a question “Did any of the following job-related demands affect your performance at work post-injury”. This question was followed by lists of stressors from the social environment (four stressors), psychological demand (four stressors) and environmental stressors (seven stressors that are grouped into physical and energy-related stressors in workplace environment), S1 File. The response was captured as ‘yes’ or ‘no’ options for presence of each stressor. The responses were categorized into *physical stressors* (i.e., barriers related to the workstation, working materials, and mobility), *stressors from energy* (i.e., discomfort related to lighting, temperature and noise), *social stressors* (i.e., barriers related to information, feedback and relationship) and *psychological stressors* (i.e., demands related to the flow, control and speed of work).

### Ethical considerations

The research protocol was reviewed, and ethical clearance was obtained from the Queen’s University Health Sciences and Affiliated Teaching Hospitals Research Ethics Board (HSREB)_ TRAQ #6033129. Further, ethical clearance was obtained from the College of Health Science Institutional Review Board (IRB), Addis Ababa University. Permission was granted at the study setting, AaBET Hospital, to access medical records for data extraction. Informed verbal consent was obtained from each study participant right before the telephone interviews. Once the data collector connected to the study participant, data collectors confirmed the participant’s language of preference and provided information about the study, and eligibility would be checked. After the participant was deemed eligible, informed verbal consent was obtained using the Letter of Information (LOI) and Informed Consent Form (ICF), followed by interviews at their preferred date and time. Whenever the individual was not willing to give consent to participate in the study, data collectors ended the conversation with thanks, and the case was recorded as ‘refused to participate.” Finally, data collectors filled and submitted a verbal consent log documentation with a checkbox and signature. All of these procedures and forms were approved with the research protocol as ethical considerations.

### Data analysis

The data from HMIS were in Excel, and the data from charts entered in Excel, while interview data were entered into the EpiData program by a data clerk. The first author subsequently exported all the data to SPSS software and merged it into a single file, using a unique identification code. The first author then checked and cleaned the data, and conducted the data analysis, using multiple approaches—logistic regression and survival analysis.

Logistic regression [38] was employed to explore factors associated with the RTW status (yes/no) of injury survivors at one year post injury. The process of choosing independent variables was informed by the Systemic, Ecological Model for Rehabilitation Counselling, with a biopsychosocial lens [20, 21]. A series of bivariate regressions were conducted to choose variables for the multivariable regression. All variables with *p*-value <0.2 in the binary regression were moved to the multivariable model so that variables relevant to the outcome were not missed. Multicollinearity was assessed using the variance inflation factor (VIF). Also, Omnibus test was used to explore the significance of differences between groups and Hosmer-Lemeshow goodness of fit tests was used as model diagnostics. The multivariable logistic regression analysis was performed with iterative processes to find the best model fit. The analysis outputs are presented in adjusted odds ratio (AOR), with 95% CI and a cut point for statistical significance was determined at *p*-value ≤ 0.05.

Furthermore, survival analysis [39] was carried out to investigate the variation of time (weeks) to RTW within one year after injury. The Kaplan-Meier survival curve was generated for each categorical variable, and the log-rank test was used to assess the difference in time to RTW between groups. Multivariable Cox regression was used to identify those variables with p-value < 0.2 to include in the Cox regression model. The selection of variables that could determine the time to RTW was with the assumption that ‘no change happened on the variable after the injury occurred’. The association between the time to RTW and covariates was assessed by adjusted hazard ratios at 95% CI and p-value of ≤ 0.05. The distribution of the data was not symmetrical; hence, the median time was found to be appropriate to estimate the cases of survival time, at which 50% of the injury survivors were successful in RTW. An injured employee who did not return to work within one year after the injury or whose RTW outcome was not known one year after the injury (the end of the study) was censored. The analysis results are presented with narrative descriptions, followed by tables.

## Results

### Population characteristics

As previously noted, this analysis is based on 251 injury survivors who were interviewed and completed the full interview. Of these, 75% were young adults (age </ = 39 years), 78% were male, 78% were urban residents, 41% were injured by road traffic collisions, 51% had fractures at various body parts, 65% experienced work-related injury, 53% were discharged from hospital within a week, and 87% were in manual occupations.

### Return to work outcomes and associated factors

**Fig 1:**
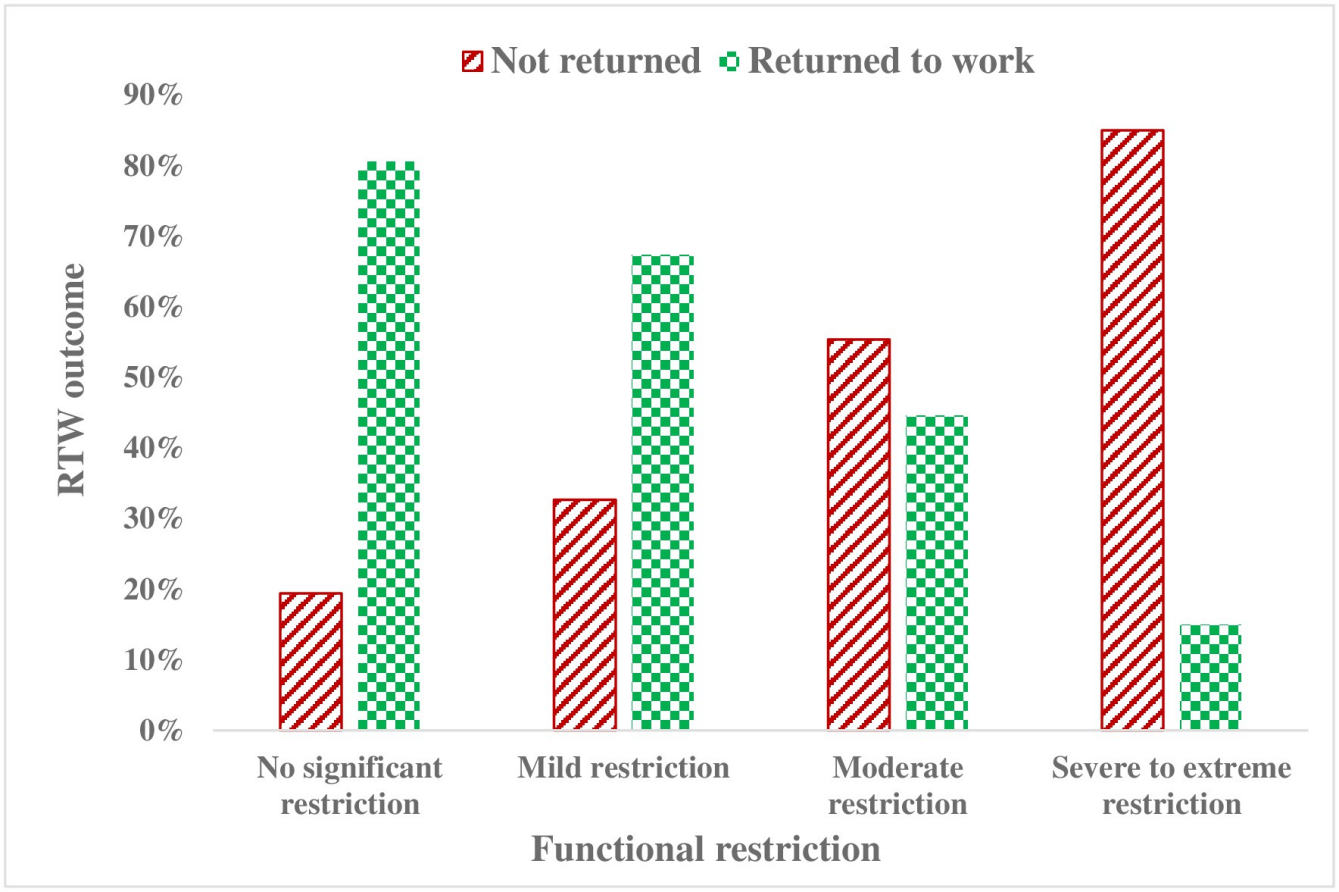
RTW by the level of functional restriction of injury survivors, 2022 (n = 251).

One hundred and forty-seven (59%) of participants had returned to work one year post-injury. Data revealed that traumatic injury significantly affects the functional status of survivors and consequently jeopardizes their employment outcome. The finding indicates that one year after traumatic injury, 61% of injury survivors remained with some form of functional restriction. This analysis indicates that as the level of functional restriction increased the RTW success of injury survivors was reduced, Fig 1.

Multiple factors influenced the RTW outcomes of injury survivors. The logistic regression analysis revealed six independent variables that are significantly associated with the likelihood of injury survivors’ RTW after injury. These factors include personal factors (i.e., having the motivation to RTW), injury and health-related factors (i.e., short hospital stay, no disability, and no chronic illness) and employment-related factors (i.e., being in an administrative position and received compensation), Table 1.

**Table 1:** Factors associated with the return to work success of injury survivors 12 months post-injury, 2022 (n = 251).

Variables	Category	Total	RTW status (n)		AOR (95% CI)	*P-value*
			Yes, n (%)	No, n (%)		
**Biological Sex**	Male	196 (78%)	120 (61%)	76 (39%)	1.27 (0.56–2.88)	0.572
	Female	55 (22%)	27 (49%)	28 (51%)	1	
**Age**	</ = 39 years	187 (75%)	115 (61%)	72 (39%)	1.62 (0.72–3.65)	0..243
	>39 years	64 (25%)	32 (50%)	32 (50%)	1	
**Educational status**	No formal schooling	33 (13%)	12 (36%)	21 (64%)	1	
	Primary	91 (36%)	50 (55%)	41 (45%)	2.32 (0.74–7.22)	0.147
	Highschool	93 (37%)	59 (63%)	34 (37%)	2.90 (0.94–9.00)	0. 064
	College and above	34 (14%)	26 (76%)	8 (24%)	1.96 (0.40–9.90)	0. 414
**Residence**	Urban	196 (78%)	122 (62%)	74 (38%)	0.68 (0.30–1.55)	0.359
	Rural	55 (22%)	25 (45%)	30 (55%)	1	
**Motivation to RTW**	Have motivation	181 (72%)	117 (65%)	64 (35%)	3.50 (1.61–7.50)	**0.002** *
	No motivation	70 (28%)	30 (43%)	40 (57%)	1	
**Types of injury**	Traumatic brain injury	70 (28%)	41 (59%)	29 (41%)	1	
	Fracture	128 (51%)	67 (52%)	61 (48%)	1.02 (0.10–14.64)	0.988
	Injuries of penetrative, soft tissue, & strain/sprain/dislocation	53 (21%)	39 (74%)	14 (26%)	1.73 (0.11–28.20)	0.699
**Body parts injured**	Spine & bones of the trunk	15 (6%)	10 (67%)	5 (33%)		1
	Head	74 (29%)	30 (41%)	44 (59%)	0.120 (0.01–1.31)	0.082
	Extremities	135 (54%)	60 (44%)	75 (56%)	0.441 (0.02–10.52)	0.613
	Chest & internal organs	10 (4%)	2 (20%)	8 (80%)	0.240 (0.03–1.69)	0.152
	All others	17 (7%)	2 (12%)	15 (88%)	0.420 (0.03–6.10)	0.525
**Length of inpatient stay**	Up to one week	132 (53%)	97 (73%)	35 (27%)	4.20 (2.10–8.50)	**< .001** *
	More than one week	119 (47%)	50 (42%)	69 (58%)	1	
**Disability status a year post-injury**	Have no restriction	98 (39%)	79 (81%)	19 (19%)	4.44 (2.10–9.60)	**< .001** *
	Have some level of restriction	153 (61%)	68 (44%)	85 (56%)	1	
**Have chronic illness a year post-injury**	No	167 (67%)	106 (63%)	61 (37%)	2.31 (1.14–4.70)	**0.020** *
	Yes	84 (33%)	41 (49%)	43 (51%)	1	
**Compensation for injury**	Received	40 (16%)	27 (68%)	13 (33%)	3.10 (1.22–7.73)	**0.017** *
	Didn’t receive	211 (84%)	120 (57%)	91 (43%)	1	
**Types of jobs (administrative/manual)**	Administrative	32 (13%)	28 (88%)	4 (13%)	5.32 (1.11–25.78)	**0.038** *
	Manual	219 (87%)	119 (54%)	100 (46%)	1	
**Pre-injury employer**	Government/Public	32 (13%)	26 (81%)	6 (19%)	1.92 (0.36–10.23)	0.445
	Private	127 (51%)	72 (57%)	55 (43%)	0.93 (0.50–1.94)	0.844
	Self family	92 (37%)	49 (53%)	43 (47%)	1	
**Pre-injury workplace size**	Medium and large enterprises	50 (20%)	35 (70%)	15 (30%)	1.91 (0.70–5.21)	0. 207
	Micro and small enterprises	201 (80%)	112 (56%)	89 (44%)	1	
**Have physical stressors at work**	No	110 (44%)	59 (54%)	51 (46%)	0.62 (0.31–1.23)	0. 168
	Yes	141 (56%)	88 (62%)	53 (38%)	1	

*Variables with significant *p*-value (<0.05)

Concerning injury and health-related factors, length of hospital stay—a measure of injury severity—was one of the factors with the strongest association with RTW after injury. Injury survivors with shorter length of inpatient stay (discharged within a week) had a four times higher probability of RTW after injury (AOR = 4.20; 95% CI: 2.10–8.50), with *p* ≤ 0.001. Moreover, the level of functional restriction (disability status) was another factor with significant association to the RTW status of participants. Injury survivors who have no residual functional restriction due to the injury show four times higher probability to resume work within a year, compared to those with some level of residual impairment (AOR = 4.44; 95% CI: 2.10–9.60), with *p* ≤ 0.001. Further, living with chronic illnesses was strongly associated with the outcome, with participants who had no chronic illness show higher probability of RTW after injury compared to those with at least one type of chronic illness (AOR = 2.31; 95% CI: 1.14–4.70), with *p* = 0.020.

Moreover, individuals who received compensation for the injury showed three times higher probability of RTW compared to non-recipients (AOR = 3.10; 95% CI: 1.22–7.73), with *p* = 0.017. Among the factors associated with participants’ employment characteristics, injury survivors who were in administrative positions (in white collar jobs) showed more than five times higher likelihood to RTW after injury compared to manual workers (in blue collar jobs) (AOR = 5.32; 95% CI: 1.11–25.78), with *p* = 0.038. From personal factors, individuals who had motivation to resume work even before their full recovery were three times more likely to be found returned to work one year after injury (AOR = 3.50; 95% CI: 1.61–7.50) with *p* = 0.002, Table 1.

### Predictive factors of time to return to work after injury

**Fig 2:**
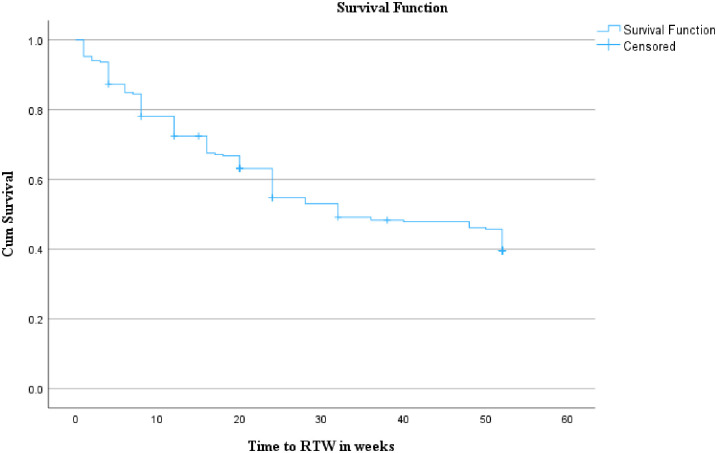
Kaplan-Meier survival function of time to RTW after traumatic injury, 2022 (n = 251).

In this study, the time to RTW after injury was captured in weeks, with a range from 0 (less than a week) to 52 weeks. As mentioned earlier, our data are not sufficiently symmetrical to rely on the mean. The median indicates that 50% of the returned injury survivors resume work within 32 weeks (95% CI: 23.40–40.50), Fig 2.

**Fig 3:**
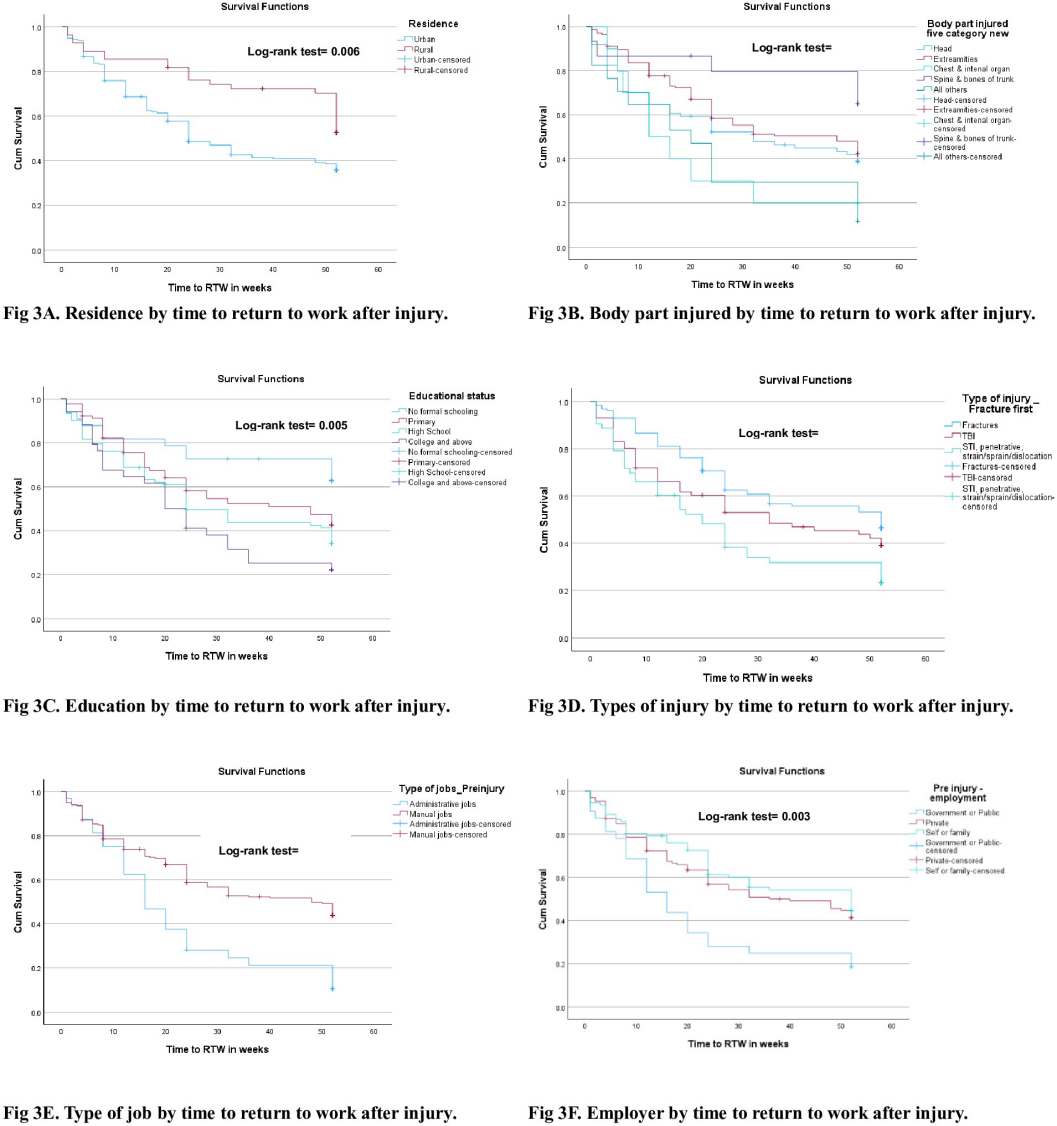
Kaplan-Meier survival function of important variables that show difference in time to RTW after traumatic injury, 2022 (n = 251). (A) STI = Soft Tissue Injury. (B) TBI = Traumatic Brain Injury.

The time it took for individuals to resume work after injury was influenced by several factors. A series of Kaplan-Meier analyses were run to compare groups in relation to time to RTW after injury. The Kaplan-Meier analyses identify a high likelihood (*p*-values ≤ 0.05) of early RTW among participants with the highest educational status, who live in urban settings, work in administrative positions, work in public sectors and who were injured on the chest, internal organs or skin, Fig 3.

Further, univariate Cox regression was conducted to identify factors with *p*-value ≤ 0.02 to move them to the Multivariable Cox model. The multivariable model revealed that the time to RTW after injury was determined by the number of days from injury to admission and the types of injury. The probability of RTW was earlier among survivors who had access to healthcare services immediately (the same day of the injury) than those who arrived at the hospital a day after and beyond (AHR = 1.54; 95% CI: 1.05–2.25), *p* ≤ 0.026. Further, participants who were admitted due to injuries that penetrated internal organs or on soft tissues or strain/sprain/dislocation had a better probability of RTW earlier than survivors of fracture at various parts of the body (AHR = 1.81; 95% CI: 1.20–2.80), *p* = 0.007, Table 2.

**Table 2:** Predictive factors for time to RTW after injury, Multivariable Cox proportional hazard model, with an adjusted hazard ratio (AHR), 95% CI and p-value (n = 251).

Variables	Categories	Frequency	AHR (95% CI)	*P-value*
**Biological sex**	Male	196	1.18 (0.75–1.86)	0.477
	Female	55	1	
**Age**	</ = 39 years	187	1.22 (0.80–1.90)	0.361
	> 39 years	64	1	
**Education**	No formal schooling	33	1	
	Primary	91	1.34 (0.68–2.65)	0.396
	High School	93	1.67 (0.86–3.30)	0.130
	College and above	34	1.50 (0.67–3.31)	0.327
**Residence**	Urban	196	1.55 (0.95–2.54)	0.078
	Rural	55	1	
**Type of injury**	Fracture	128	1	
	Traumatic brain injury	70	1.29 (0.86–1.95)	0.223
	Injuries of penetrative, soft tissue, & strain/sprain/dislocation	53	1.81 (1.20–2.80)	**0.007** *
**Causes of injury**	Work-related	164	1.20 (0.77–1.73)	0.482
	Not work-related	87	1	
**Days from injury to hospital**	On the same day	147	1.54 (1.05–2.25)	**0.026** *
	After a day or more	88	1	
**Type of jobs**	Administrative	32	1.65 (0.82–3.31)	0.163
	Manual labour	219	1	
**Pre-injury Employer**	Government	32	1.18 (0.57–2.50)	0.663
	Private	127	0.95 (0.62–1.50)	0.807
	Self or family	92	1	

*Variables with significant *p*-value (<0.05).

## Discussion

The aim of this study was to examine factors that are associated with the RTW outcomes and determinants of time to RTW after injury. We would like to highlight that in this study, RTW has been examined as a binary outcome, such that if a participant had returned to work with partial work hours and/or wages, it would still be counted as ‘successful RTW’. The study revealed important personal factors, injury and health-related characteristics and employment conditions associated with RTW success after injury. Again, the type of injury and number of days elapsed from incident of injury to healthcare access show significant association of these variables with time to RTW after injury. The discussion summarizes the findings into four meaningful themes.

### Injury related factors—Severity, type, and access to healthcare are vital in trauma care as they influence post-injury work reintegration

In this study, the strongest predictor of RTW was determined to be length of hospital stay—the proxy measure for injury severity. Injury survivors with shorter periods of hospital stay (discharged within a week) showed an increased probability of RTW compared to those admitted for a prolonged time. Our finding is similar to that of previous studies [30, 40, 41], in which severity of injury was reported as a strong predictor for RTW. We speculate that prolonged inpatient admission could mean the injury was severe and/or the healthcare services are not able to effectively ensure fast recovery to discharge patients from the hospital. Severe injury could lead to serious functional impairment, which has implications on the probability of an individual’s RTW. Although the severity of the injury is one of the most reported predictors of RTW success [42, 43], studies in Australia [44] and in Germany [45] reported the opposite, with a justification that other factors like personal (psychological) and healthcare/rehabilitation related factors could severely impact RTW more than the initial physical injury. This argument may work in the case of these two studies because the Australian study [44] included only patients due to road traffic collisions, and the German study [45] reported on patients in an intensive care unit (ICU), where the injury severity may have been less variable. Participants of the current study, however, were from the general injury population, with a wide range of injury types and severity. Most importantly, the length of inpatient admission could serve as a better proxy indicator for injury severity, particularly for low-income settings—which is the case of this study. Healthcare providers in low-resource settings have limited resources. Hence, they may need to focus on the most severely injured individuals while mildly injured patients may potentially be discharged and resume work right away. Nevertheless, we want to note that prolonged hospital stays may not happen only because of the injury severity, as several other personal and institutionally related factors can influence patients’ overall health and recovery and thus prolong the hospital stay and delay resuming work.

Our further analysis indicates that prolonged time to RTW was prevalent among participants who had delayed access to healthcare (a day after injury or beyond) and among survivors who experienced fracture versus injuries of penetrative, soft tissue, strain/sprain/dislocation. Fractures are often associated with prolonged in-hospital care and lead to job-related functional impairments; hence, patients need extended support services to alleviate residual sequelae [42, 46]. Also, fractures on various parts of the body are common when traumatic injuries happen and often lead to ongoing pain and impairment [47] that can negatively affect employment outcomes compared to soft tissue injuries and strain/sprain/dislocation [47], which is the case in this study. Further, complex and multilayered factors that could delay patients’ access to healthcare after injury were reported in African studies [48, 49], including personal and cultural contexts (e.g., lack of awareness and poor perception of healthcare), physical barriers (distance and transport to healthcare) and financial barriers related to healthcare and transport costs. Delayed access to healthcare after injury was reported as one of the major causes of injury-related deaths [50] and one of the key factors for prolonged hospital stay and associated implications [51] that could include delay in resuming work after injury, as this is the case in the current study. A holistic approach with a continuum of care at acute, in-patient and community integration, with critical consideration to prioritize patients by the severity and types of injury [52], could improve the residual work disabilities and success in RTW for survivors.

### Functional and health states of injury survivors implicate RTW outcomes

Our findings revealed a statistically significant association between functional restriction (disability) and RTW success after injury. Traumatic injury can impact individuals’ overall health and often leave survivors with residual functional restriction [53, 54]. Data presented in Fig 1 shows that the level of functional restriction and the probability of RTW after injury are inversely related to each other. It is likely, as in other studies [52, 53], that injury led survivors to reduced functional status that negatively impacted employment success. This could be because individuals with disability have poor capacity to self advocate for necessary supports and workplaces may not have arrangements to support survivors with residual impairment returning to work, particularly in Ethiopia where there is a lack of professional and policy guidance with a focus on RTW.

Furthermore, this study shows a higher probability of RTW among individuals who reported being free from chronic illness at the time of the survey versus those with at least one chronic illness. This finding aligns with earlier studies that reported increased success in RTW in the absence of chronic illness [44]. Also, the presence of comorbidity was documented as a prognostic factor for negative functional outcomes after injury [55, 56]. We speculate that chronic illnesses could significantly influence survivors’ recovery and function by imposing an additional burden on injury survivors and workplaces, especially if these diseases are not controlled and/or necessary supports are not arranged in relation to RTW [57].

### Employment characteristics hold key capabilities to enhance RTW success after injury

One of the strongest determinants of RTW was the type of preinjury work. Those in administrative positions (white collar—managers, teachers, and businesspersons) show a higher likelihood of resuming work after injury versus those in manual occupations (blue collar—daily labourers, outdoor workers, and machine operators). Literature in the field has also reported similar findings [28, 41, 58]. Physically demanding tasks are predominant in blue-collar positions, which may delay the RTW of survivors from major injuries, particularly if survivors have residual functional restriction [28, 52]. Our data also reflect this trend, as most white-collar workers had contractual work agreements. Therefore, employers may become responsible for arranging support mechanisms. In Ethiopia, low-skilled and poorly educated workers, youth, and women are overrepresented in informal jobs, small businesses and self-employment [59, 60]. These are often precarious forms of employment. Hence, RTW success after disabling injury may be jeopardized due to their existing vulnerability, and the consequences could have ripple effects on families who were dependent on the injured breadwinner [61]. Earlier studies reported better RTW success among individuals working in larger companies [28, 58, 62]. This could be due to the capacity of larger employers to arrange accommodations, create supervisory support systems and offer alternate duties for injured workers [63].

In this study, the Kaplan-Meier analysis results revealed that injury survivors who worked in the public sector returned to work earlier than private sector employees and self-employed ones. We speculate that family-owned businesses are often smaller companies, which can reduce the commitment to RTW after injury as other family members may take on the responsibility of the injured, resulting in less financial pressure on the injured person—removing what could have been the driving force to RTW soon after injury.

Further, this study identified that individuals who received monetary compensation for the injury were more likely to RTW after injury. The result is in line with another study that reported positive associations [64], which is in contrast to the findings of earlier studies conducted in developed economies where compensation can be associated with workers taking more time for recovery [65, 66]. The discrepancies could be because of the differences in policy frameworks, social constructs and nature of the compensation measurement, as there is lack of conclusive agreement on what and how to measure compensation [67]. We speculate that recipients of monetary compensation may develop a sense of belongingness or justice [41] that could encourage them to resume their jobs. Overall, our sample is from a general acute trauma population who are with a broad nature of injury causes and diverse employment conditions. This could make the compensation issue complex, particularly for those employed in self/family business, informal/uninsured workers and when the injury is not compensable such as self-injury, by a family/relative/friend, or assault by an unknown person. In line with this, it is possible that individuals may not be aware of their right or the process to claim compensation for their injury. Further, the available legal frameworks of the social security system may not cover the individual’s type of employment, or their employer may not have insurance coverage [23, 26].

Despite the fact that employers are required by the law to provide employment protection schemes for their workers, in Ethiopia, there is a limited scope of legal frameworks available to guide the employment injury compensation process [23]. We also have limited data available through this study regarding the compensation characteristics and reasons for the low compensation rate (16%). The association of compensation with the RTW outcomes is not sufficiently documented in the Ethiopian context. Hence, further research about the compensation features and implementation system is crucial to understand its influence on the RTW outcomes of injury survivors.

### Personal background has vital role on the RTW outcome after injury

Personal values and desire to engage in occupations are vital in the post-injury RTW. An individual’s motivation to return to work is one’s desire to engage in a certain behaviour and actions during the recovery after work disability [68, 69]. The current study revealed that injury survivors who were motivated to RTW—despite their poor recovery or work capacity—had higher probability of RTW. Individual’s motivation could be influenced by one’s capacity and the value attached to the work, and thus can play a crucial role in determining employment outcomes after injury [70, 71]. Injury survivors behave in a certain manner in the RTW trajectory, which may be influenced positively by their motivation and negatively by poor functional capacity and barriers in the RTW process [68, 69]. This process could be connected to the concepts of the Self Determination Theory [68, 72] which features autonomy, competency and relatedness of the individual within their social system. This requires in-depth exploration to understand better how intrinsic and extrinsic factors relate individuals’ motivation with RTW after work disability, particularly within a context like Ethiopia where formal RTW support systems are not available.

Furthermore, the Kaplan-Meier analysis showed that RTW after injury was earlier among urban residents, those with higher educational status and with professional/vocational skills by training. This may imply that urban residents have better access to rehabilitation services, as was reported earlier [73], which could enhance their RTW success. The data also indicates that urban residents had better educational status and professional/vocational skills by training than participants from rural settings. In this analysis, residence, educational status and professional/vocational skills were not statistically significant in the Cox proportional hazard model. Even so, earlier studies of RTW in Ethiopia [14] and other countries [30, 74] reported the benefit of professional training and better education for RTW success. We speculate that individuals with specialized skills/professions could have better potential to be in stable jobs that can offer access to appropriate support during post injury transition to RTW. Education gives access to administrative (non-manual) jobs, and these conditions may provide power for individuals to negotiate and advocate for themselves, which could help them gain access to needed financial, healthcare and social supports [28, 58]. In addition, administrative jobs may have better flexibility to accommodate the shifting needs seen after the injury. Overall, promoting work’s economic and health benefits is crucial to engage key stakeholders (employers, injury survivors, care providers and policymakers) in actions to enhance the RTW’s success after injury.

## Limitations

This study was conducted with participants drawn from a single setting and provided information concerning patient outcomes within this context. Also, the final participants were limited to only 251 survivors, which might have influenced some of the results. Hence, the findings may not generalize to the larger population. Furthermore, despite their likely relevance to RTW rates post-injury, some crucial factors from injury characteristics and employment context did not emerge as significant predictors of RTW. This may be because of the broad nature of the population studied. Another potential reason could be our need to collapse the response options of several variables to facilitate analysis. In collapsing variables, details that may have been relevant were lost. For example, variables capturing injury characteristics, such as the mechanism of injury and body part injured, were documented in detail from the medical records, while variables capturing occupational contexts, such as employment sectors and organization size, were collected with open-ended questions during the interview. In both cases, the way in which these variables were grouped into meaningful categories may have influenced the potential to identify differences in RTW outcomes. However, the study design allows us to understand the practically important factors that are associated with the RTW success of survivors and time to RTW following major injuries, caused by a wide range of mechanisms and after treatment within one healthcare setting. Not all of the identified factors are necessarily modifiable, but the findings identify at-risk groups who could benefit from targeted interventions tailored to their needs and characteristics to improve their RTW outcome. Further, variables such as health conditions other than chronic illnesses, ass well as contextual factors such as quality of care received, access to rehabilitation services and occurrence of subsequent injuries, none of which were examined in this study, may have influenced the RTW outcomes. Future research may consider these factors for a better understanding of contexts. Also, we measure factors one year post-injury; hence, the result could be different if the measurements were conducted at a different time frame after injury, such as right after an injury, after three months or six months. More research is needed to explore the RTW conditions of injury survivors in the Ethiopian and other low-income contexts.

## Conclusion

This study revealed major contributors to the likelihood of RTW success after injury, including *personal factors* (i.e., motivation to RTW while on recovery), *injury and health-related factors* (i.e., short hospital stay, no functional restriction, and absence of chronic illness), and *employment-related factors* (i.e., being in an administrative job preinjury and access to compensation for injury). In addition, participants returned to work earlier if they received access to healthcare services immediately after injury and survived injuries of *penetrative*, *soft tissue and strain/sprain/dislocation*. It may be important to initiate RTW interventions right after injury—while in the hospital and along the recovery process—with support extended after hospital discharge. In order to enhance injury survivors’ timely occupational engagement, critical action is required for timely RTW intervention with attention to the identified factors influencing the RTW outcomes. Not all of the factors identified are modifiable; hence, interventions may consider them as issues to be addressed in actions such as identifications of individuals’ needs and areas of intervention to support their RTW. Policymakers, trauma care providers and researchers need to make RTW a central value in the planning and provision of support during the recovery process of injury survivors.

## Supporting information

S1 File Telephone survey instrument English. (PDF)

S1 Dataset (XLSX)

S1 Checklist Chart extraction checklist. (DOCX)
