# Supplementary material for: Resuming work roles after injury in a low-income context: Multiple factors influencing the return to work outcomes
Source: PLoS One. 2024 Oct 23;19(10):e0308816. doi: 10.1371/journal.pone.0308816 (PMC11498707; doi:10.1371/journal.pone.0308816)
Supplement: S1 File — (PDF) [file pone.0308816.s001.pdf]

Unique Identification Code (UIC): ________________________

Data Collection Instrument for Telephone Interview among Injury Survivors

Date of Interview: _____________

Name of data Collector: __________________________

Individuals involved in the interview: a) Injury survivor only b) Survivor plus an assistant

Language used during the interview: a) Amharic b) Afaan Oromo

I will try read the questions to you, clearly. But feel free to stop me if my voice or the question itself is not clear for you so I can adjust and repeat questions.

Let us start with some background information.

Ser. No.	Sociodemographic Information	Response Options	Instruction/Skip
1.	What is the highest grade that you completed in school?	a. Can't read and write<br>b. Elementary (1-4th grade)<br>c. Junior (5th- 8th grade)<br>d. Highschool (9th-12th grade)<br>e. College/University (undergraduate/certificate)<br>f. Graduate study	
2.	What is your religion?	a. Orthodox<br>b. Muslim<br>c. Protestant<br>d. Catholic<br>g. Others (specify): __________	
3.	What is your marital status?	a. Single / never married<br>b. Married<br>c. Divorced / separated<br>d. Widow/er	
4.	How many family members are living with you in the same house?	______________	
5.	Do you live in an urban or rural area?	a. Urban b. Rural	

Unique Identification Code (UIC): ________________________

**Injury Related Information**			**Instruction/ Skip**
6.	Did the traumatic injury happened while you are on activities related to your work?	a. Yes<br>b. No	
7.	How long did you stay admitted in the hospital because of the injury?	__________ days	
8.	Have you ever received rehabilitation treatment or support for effects associated with the traumatic injury?	a. Yes<br>b. No	If No, skip to Q#10
9.	If yes to above, from where did you receive rehabilitation service/support and what type of support have you received?<br>**Note**: More than one option is possible.	a. From a rehabilitation institution: ________________________<br>b. From traditional medicine: ________________________<br>c. From a religious institution: ______________________<br>d. Others (specify): _________ ________________________	

10. Tell me if there is any assistive device you are using as a result of the injury:
    a. I am not using, because I do not need it
    b. I am not using, because I don't have access or capacity
    c. Yes, I am using: Type/s: ______________________; ________________________

11. We would like to know about any potential residual impairments. I am going to read questions and you will choose one that best describe about your current level of difficulty in functioning domains. There are five options, ‘0-4, these include:
    0 = I have no difficulty
    1 = Mild difficulty
    2 = Moderate difficulty
    3 = Severe difficulty
    4 = Extreme difficulty

Unique Identification Code (UIC): ________________________

Domains	How difficult is it for you to do the following?	No difficulty	Mild difficulty	Moderate difficulty	Severe difficulty	Extreme difficulty
**Cognition**	Concentrating on doing something for 10 minutes?	0	1	2	3	4
	Learning a new task, for example, learning how to get to a new place?	0	1	2	3	4
**Mobility**	Standing for long periods, 30 min?	0	1	2	3	4
	Walking a long distance such as 1km or equivalent?	0	1	2	3	4
**Self-care**	Washing your whole body?	0	1	2	3	4
	Getting dressed?	0	1	2	3	4
**Interpersonal relationships**	Dealing with people you don't know?	0	1	2	3	4
	Maintaining a friendship?	0	1	2	3	4
**Activities of daily living**	Taking care of your day-to-day activity, e.g., housework, leading the family, childcare, managing cattle's etc.	0	1	2	3	4
	Managing your day-to-day work/study?	0	1	2	3	4
**Participation**	Joining in community activities, for example, how often do you find yourself at religious or social event/gatherings, just like any other person?	0	1	2	3	4
	How much have you been emotionally affected by your health problems?	0	1	2	3	4

Unique Identification Code (UIC): ________________________

12. Do you have any of the following chronic health conditions confirmed by a health professional? I will read the lists for you and indicate if they are 'Yes or No'.

**Health Conditions**	**Response**
12.1 Hypertension	☐ Yes ☐ No
12.3 Heart disease	☐ Yes ☐ No
12.5 Angina (chest pain)	☐ Yes ☐ No
12.7 Arthritis (rheumatoid and osteoarthritis)	☐ Yes ☐ No
12.9 Osteoporosis	☐ Yes ☐ No
12.11 Neurological disorders	☐ Yes ☐ No
12.13 Peripheral vascular disease	☐ Yes ☐ No
12.15 Depression	☐ Yes ☐ No
12.17 Anxiety or panic disorders	☐ Yes ☐ No
12.19 Diabetes (type I or II)	☐ Yes ☐ No
12.21 Degenerative disc disease (back/spinal pain)	☐ Yes ☐ No
12.23 Stroke	☐ Yes ☐ No
12.25 Unspecified pain	☐ Yes ☐ No
12.27 Dementia	☐ Yes ☐ No
12.29 Gastrointestinal disease	☐ Yes ☐ No
12.31 Asthma	☐ Yes ☐ No
12.33 Chronic respiratory/lung disease (COPD)	☐ Yes ☐ No
12.35 Skin problem	☐ Yes ☐ No
12.37 Any allergy (specify): ________________	☐ Yes ☐ No
Do you have the following sensory impairments (sight and hearing); if yes, indicate if this is before or after the injury?	
12.40 Reduced visual acuity or complete inability to see	☐ Yes ☐ No
12.42 Reduced hearing capacity/complete inability to hear	☐ Yes ☐ No

Unique Identification Code (UIC): ________________________

**Employment Related Information**			**Instruction / Skip**
13.	Are you currently returned to any work/job?	a. Yes<br>b. No	
14.	How do you describe your current status to RTW? Listen to what I am saying and choose one of the statements that best describe your current state.<br>***Note:- ask questions:***<br>'a-c' for off-work groups<br>'d-f' for working groups	**<u>For off-work groups</u>**<br>a. I am not thinking about starting work<br>b. I have started to think about returning to work, but has no concrete plan<br>c. I have started to seek information and have concrete plans for RTW (preparation)<br>**<u>For on-work groups</u>**<br>d. I have returned to work, but struggling to maintain (uncertain maintenance)<br>e. I have returned and found good strategy to manage the work (active maintenance)<br>f. I have returned and attempting to promotion/improvement (advancement)	
15.	How do you rate your current work ability compare to your ability before the traumatic injury?	0 = I can't work at all<br>1 = Very low work ability<br>2 = Low work ability<br>3 = Medium work ability<br>4 = High work ability<br>5 = Very high work ability	If currently working, skip to Q#17

Unique Identification Code (UIC): ________________________

16.	If not working currently, have you ever returned to work after the injury?	a. Yes<br>b. No (never returned)	If b, skip to Q#18
17.	If you had ever returned to work after the injury, when did you first resume work after the injury?	_________________ (Weeks)	
18.	If you are not working currently, why are you not on the job?	a. Serious disability or illness<br>b. Unable to find job<br>c. Failed to cope up with job-related demands after the injury:<br>d. Retirement<br>e. Pregnancy or maternity/parental leave<br>f. Studying or on training<br>g. Caring for child/ren of own/relative(s)<br>g. Personal choice<br>h. Other (specify): ___________	
19.	Have you experienced any impact on your job due to the current national situation or related to COVID-19?<br>If yes, please let me know:	a. No, I did not have/experienced any impact<br>b. Yes, in connection with current national conditions: _____________________<br>c. Yes, in connection with the COVID-19: ______________________________	
20.	Do you have any vocational skill set or profession with formal education or training?	a. Yes: tell me what it is:<br>Professional: ________________<br>Vocational: ________________<br>b. No, I did not receive any formal education or vocational training	

Unique Identification Code (UIC): ________________________

**Instruction for data collectors:**<br>If returned, ask <u>both the pre and post injury</u><br>If not returned, ask <u>only the preinjury</u>		**Preinjury job**	**Current job**	**Same/different/ remark**
21.	What is the type of job you do?			
22.	Where/in which organization/sector do you work?			
23.	What is your employment relationship?<br>1. Definite/temporary: contractual for a specific period<br>2. Indefinite/permanent<br>3. Daily labour/seasonal: without contract/unstable jobs<br>4. Self-employed/family business			
24.	Approximately how many people work in your workplace?<br>a. <10 workers<br>b. 10-30 workers<br>c. 31-100 workers<br>d. >100 workers: _____________			
25.	How long have you worked in that job?	__________	__________	
26.	How many hours do you work per week?	_____hrs/wk	_____hrs/wk	
27.	How much do you earn from the employment/job monthly?	_______birr	_______ birr	

<u>Note for data collector</u>: If the participant has <u>not returned to work currently</u>, skip to Q#29.

28. Where do you get supports in relation to work after the injury?

**Sources of support**		**Type of support**
○ Co-workers	☐ No ☐ Yes	_____________________
○ Management/employer	☐ No ☐ Yes	_____________________
○ Trauma care provider	☐ No ☐ Yes	_____________________
○ Rehabilitation provider	☐ No ☐ Yes	_____________________
○ Family	☐ No ☐ Yes	_____________________
○ Personal network/friends	☐ No ☐ Yes	_____________________
○ Spiritual leader/groups/institution	☐ No ☐ Yes	_____________________

○ Other (specify): ____

Unique Identification Code (UIC): ________________________

29. Dis any of the following job-related demands affected your performance at work post injury?

- **Pressure/demand from physical Environment:**
  1. Work space/station inconvenience ☐ No ☐ Yes
  2. Discomfort with material at work ☐ No ☐ Yes
  3. Inconvenience for movement ☐ No ☐ Yes
  4. Discomfort with light/illumination ☐ No ☐ Yes
  5. Noise pollution ☐ No ☐ Yes
  6. High temperature ☐ No ☐ Yes
  7. Low temperature ☐ No ☐ Yes
  8. Other (specify): ____

- **Psychological demand:**
  9. Poor work controls ☐ No ☐ Yes
  10. Unable to cope with work speed ☐ No ☐ Yes
  11. Inconvenience with work process/flow ☐ No ☐ Yes
  12. Poor control on rest/break periods ☐ No ☐ Yes
  13. Other (specify): ____

- **Demand from social environment:**
  14. Unable to get support when needed ☐ No ☐ Yes
  15. Poor relationship with supervisor/employer ☐ No ☐ Yes
  16. Poor access to information ☐ No ☐ Yes
  17. Dissatisfied with promotion/feedback ☐ No ☐ Yes
  18. Other (specify): ____

30. Do you prefer to go to/show up at workplace, even though your health condition is not good enough to the extent it compromises your productivity? Why?
    a. Always: ___________________________
    b. Often: ______________________________
    c. Sometimes: _________________________
    d. Rarely: ____________________________
    e. Never: _____________________________

31. Have you received any compensation or benefits for effects related to the traumatic injury?
    a. Yes
    b. Process/partially paid
    c. Not at all; why? ____

32. This is my last question; if there is anything you want to tell or ask me, I am here to listen to you before closing our conversation. Please, it is your turn.

____

Thank you for your cooperation and for your time!

Unique Identification Code (UIC): _________________________
