# Supplementary material for: Resuming work roles after injury in a low-income context: Multiple factors influencing the return to work outcomes
Source: PLoS One. 2024 Oct 23;19(10):e0308816. doi: 10.1371/journal.pone.0308816 (PMC11498707; doi:10.1371/journal.pone.0308816)
Supplement: S1 Checklist — (DOCX) [file pone.0308816.s003.docx]

**Data extraction form from patients’ chart**

|  | **Medical Registration No (MRN)** |  |
| --- | --- | --- |
|  | **Patient Name** |  |
|  | **Telephone No** | - **Patient**: +251__________________ - **Caregiver**: +251_______________ |
|  | **Date of injury: ______________; Date of arrival/admission: _______________; Date of discharge: ______________** | |
|  | **Work / occupation related issues described on the chart** | 1. Type of job: ________________________________ 2. Employment: _______________________________ 3. Other: _____________________________________ 4. No information recorded |
|  | **Body part injured**  **Note:** Circle all that apply | 1. Lower extremities 2. Upper extremities 3. Head 4. Face 5. Neck 6. Chest 7. Abdomen/internal organ/penetrative 8. Spinal cord/spine 9. Hip 10. Soft tissue injury 11. External body (skin) 12. Other specify: **______________________________________** |
|  | **Nature/type of the injury**  **Note:** Circle all that apply | 1. Deep Skull Fracture (DSF) 2. TBI 3. Fracture of spine 4. Fracture of extremities 5. Soft tissue injury/penetrative/cut, tendon, nerve, blood vessels 6. Crush injury 7. Traumatic amputation 8. Injury to internal organs 9. Injury involving multiple body 10. Injury to external body (skin) 11. Dislocation, sprain and strain 12. Other injuries of upper limb 13. Other injuries of lower limb 14. Other injuries of unspecified body, specify: **_______________________** |
|  | **Mechanism of injury** | 1. Contact with inanimate or external forces 2. Contact with hot surfaces or heat 3. Burn 4. Electrification 5. Acid injury on an external body 6. Transport accident (Road Traffic Collision) 7. Crush by external objects 8. Falling 9. Assault (fighting & stab injury) 10. Explosion 11. Bullet 12. Contact with animate external forces 13. Other, specify: _______________________ |
|  | **Referral during discharge from hospital**  Circle all that apply | 1. To home only/no referral or appointment 2. To home with future appointment 3. To another facility for further treatment 4. Home with rehabilitation referral   **Specify**: ________________________________________ |

**Date of extraction: ____________________ By: _____________________________ Sign: __________**
